# Sense of Coherence in the Trauma–Fibromyalgia Relationship: Mediation and Moderation Findings from a 2099-Participant Cohort

**DOI:** 10.3390/ejihpe16030045

**Published:** 2026-03-23

**Authors:** Wolnei Caumo, Graziele Borges Bueno, Giordano Mayer De Freitas, Guilherme Teixeira Lopes, Mariana Lentino Coelho, Julia Gomes, Caroline Leffa Venturini, Maria Eduarda Louzada, Sara Machado Peres, Iraci L. S. Torres, Andrea Cristiane Janz Moreira, Felipe Fregni

**Affiliations:** 1 Post-Graduate Program in Medical Science, School of Medicine, Universidade Federal do Rio Grande do Sul (UFRGS), Porto Alegre 90035-003, Brazil; 2 Laboratory of Pain and Neuromodulation, Universidade Federal do Rio Grande do Sul (UFRGS), Porto Alegre 90035-003, Brazilmlcoelho@hcpa.edu.br (M.L.C.); juliaggomes@hcpa.edu.br (J.G.); cventurini@hcpa.edu.br (C.L.V.); mariaeduardalouzada@gmail.com (M.E.L.); smperes@hcpa.edu.br (S.M.P.);; 3 Pain and Palliative Care Service, Hospital de Clínicas de Porto Alegre (HCPA), Porto Alegre 90035-003, Brazil; 4 Surgery Department, School of Medicine, Universidade Federal do Rio Grande do Sul (UFRGS), Porto Alegre 90035-003, Brazil; 5 Laboratório de Farmacologia da Dor e Neuromodulação, Investigações Pré Clínicas, Centro de Pesquisa Experimental, Hospital de Clínicas de Porto Alegre (HCPA), Porto Alegre 90035-003, Brazil; 6 Laboratory of Neuromodulation and Center for Clinical Research Learning, Physics and Rehabilitation Department, Spaulding Rehabilitation Hospital, Boston, MA 02114, USA; 7 Neuromodulation Center and Center for Clinical Research Learning, Spaulding Rehabilitation Hospital and Massachusetts General Hospital, Harvard Medical School, Boston, MA 02114, USA

**Keywords:** fibromyalgia, coherence sense, trauma exposure, psychosocial deprivation, resilience, mediation analysis

## Abstract

Background: The biopsychosocial model positions fibromyalgia (FM) as the result of altered pain modulation shaped by trauma, psychological vulnerability, and structural stressors. The Sense of Coherence (SOC) may be a key resilience factor explaining differences in symptom severity after similar hardships. Objectives: To evaluate whether SOC mediates and/or moderates associations between trauma-related adversity and symptom burden in FM and whether comorbidities, medication use, healthcare factors, or treatment engagement modify these relationships. Methods: In this cross-sectional study, 2099 women with FM completed an online survey assessing adversity, psychosocial factors, core symptoms, healthcare support, treatment engagement, and medication use. A theory-driven SOC composite followed Antonovsky’s model (comprehensibility, manageability, meaningfulness) using the proxy SOC composite derived from a theory-driven framework that underwent internal construct validation, including discriminant validity analyses and latent structure evaluation, but it was not benchmarked against a gold-standard SOC questionnaire. Linear regression evaluated adversity–symptom associations, SOC mediation, and moderation by SOC and medication classes. Results: Higher adversity predicted lower SOC (e.g., cumulative abuse: B = −0.25), and lower SOC predicted higher symptom burden (e.g., Fibromyalgia Impact Questionnaire (FIQ): B = −6.77), producing significant indirect effects (cognitive symptoms: 0.22; FIQ: 1.69). SOC also moderated the effects of adversity on fatigue and global impact, weakening associations at higher SOC. Comorbidities showed modest influence: hypertension had minor indirect effects (ab = 0.27), scheduled consultation produced small interactions (cognition β = −0.38 to −0.46; fatigue β = ~0.05–0.06), and stroke showed the only clinically meaningful moderation (β ≈ 4.9–5.2), all far smaller than SOC effects. Conclusions: SOC functions as a central psychosocial pathway and resilience-related factor in the association between trauma and FM symptoms. Targeting SOC-related processes may help reduce symptom burden and improve outcomes.

## 1. Introduction

Fibromyalgia (FM) is a chronic pain syndrome characterized by widespread musculoskeletal pain, fatigue, non-restorative sleep, cognitive difficulties, and impaired quality of life (Wolfe et al., 2016). Fibromyalgia affects an estimated 2–5% of the global population, with consistently higher prevalence in women (4–7%) than in men (1–2%) (Weir et al., 2006). In Brazil, the condition affects approximately 2.5% of adults (Assumpção et al., 2009). Its impact extends beyond individual suffering: a large Danish cohort showed markedly higher healthcare spending, reduced income, and increased disability pension rates among patients and even their spouses, with financial losses beginning years before diagnosis and societal costs reaching €27,000 per patient annually (Amris et al., 2024). These findings highlight FM as a multidimensional condition. Consistent with this broader impact, a UK Biobank study of 475,171 adults demonstrated that individuals with chronic widespread pain—a phenotype closely aligned with FM—had a significantly increased risk of major cardiovascular events, with hazard ratios ranging from 1.14 to 1.48 after full adjustment (Rönnegård et al., 2022).

This broader complexity aligns with the biopsychosocial model, which frames FM as a condition characterized by altered central pain modulation and multisystem dysregulation, shaped by emotional, cognitive, and social factors (Mengshoel et al., 2021). Psychological vulnerability, emotional dysregulation, and adversity contribute to greater symptom severity (Mayer et al., 2019; Häuser & Fitzcharles, 2018; Mayerl et al., 2016; Engel, 1977). However, focusing only on individual-level mechanisms may underestimate the role of structural and contextual factors. Macro-level determinants—such as economic instability, job insecurity, discrimination, environmental stressors, and barriers to healthcare—also shape vulnerability to chronic pain and worsen outcomes (Macfarlane et al., 2009; Kapos et al., 2024, Grol-Prokopczyk et al., 2025). These pressures interact with neurobiological and psychological processes across the life course, contributing to cumulative stress and dysregulation consistent with allostatic load models (McEwen & Stellar, 1993; McEwen, 1998; Danese & McEwen, 2012). Complementary frameworks, including the fear-avoidance model (Vlaeyen et al., 2016) and maladaptive learning models (Apkarian et al., 2009), further illustrate how catastrophizing, avoidance behaviors, and negative symptom interpretation reinforce pain persistence and disability in fibromyalgia.

A large body of evidence highlights trauma and adverse life experiences as key contextual factors in FM. Histories of childhood abuse, sexual violence, persistent verbal and emotional aggression, and significant interpersonal loss are more common among individuals with FM than in the general population (Häuser et al., 2011) and are associated with earlier disease onset, greater symptom severity, and higher psychological comorbidity (Kaleycheva et al., 2021). Yet not all trauma-exposed individuals develop FM, suggesting the presence of resilience-related mechanisms. Antonovsky’s salutogenic model provides an essential conceptual lens for these processes through the construct of Sense of Coherence (SOC). In conceptual terms, SOC reflects the extent to which individuals perceive life as comprehensible, manageable, and meaningful (Antonovsky, 1987), and higher SOC is associated with less emotional distress and lower symptom burden, whereas low SOC is linked to greater fatigue and psychological distress (Super et al., 2016; Aguilar-Latorre et al., 2023). Trauma exposure has been shown to weaken SOC (Schnyder et al., 2000), while SOC shapes how individuals interpret and respond to adversity, thereby influencing health outcomes (Surtees & Wainwright, 2000; Eriksson & Lindström, 2006). Contemporary evidence further shows that SOC is sensitive to cumulative psychological load and declines under sustained adversity, with lower SOC consistently associated with greater distress in chronic pain populations (Aguilar-Latorre et al., 2023), and influencing mental health trajectories across development and adulthood (Carlén et al., 2020). Together, these findings suggest that fibromyalgia severity reflects not only nociplastic mechanisms and life-course adversity, but also individual differences in how people make sense of and cope with illness-related stressors.

While the concept of SOC holds significance, research in fibromyalgia remains limited. Earlier studies often relied on small samples, heterogeneous trauma assessments, and largely descriptive approaches (Cohen-Biton et al., 2024; Kieraité et al., 2024). SOC may mediate and moderate the adversity–symptom relationship, but most evidence derives from chronic pain populations rather than FM (Aguilar-Latorre et al., 2023). Few studies have evaluated these mechanisms simultaneously across key clinical domains—pain, fatigue, cognition, sleep, and global impact—in large, well-characterized FM cohorts, while accounting for patient-level factors such as treatment engagement, healthcare access, comorbidities, and medication use. Medication use adds another underexplored layer: individuals with greater adversity and more severe symptoms are more likely to use antidepressants, benzodiazepines, hypnotics, opioids, or multiple analgesics (Häuser et al., 2014), which target partly distinct neurobiological systems involved in affect regulation, stress reactivity, sleep–wake regulation, and pain modulation, and may influence observed associations through both pharmacological effects and confounding by indication (Goldenberg et al., 2016). Conceptually, although validated SOC instruments exist, their application in large, fully online surveys of highly symptomatic individuals with fibromyalgia raises feasibility and methodological concerns, including questionnaire length, respondent burden, and overlap with symptom measures. Moreover, Brazilian Portuguese versions have shown context-dependent psychometric limitations (Scalco et al., 2020; Spadoti Dantas et al., 2014; Bonanato et al., 2009). Accordingly, and as detailed in the Methods, we operationalized SOC using a theory-driven composite (proxy) based on conceptually aligned indicators reflecting comprehensibility, manageability, and meaningfulness. This approach should be interpreted as a pragmatic and hypothesis-generating operationalization rather than a replacement for validated SOC questionnaires.

In the present study, we address these gaps using a large, community-based Brazilian cohort of women with fibromyalgia, extensively characterized across adverse life experiences, cumulative abuse indices, psychological and functional measures, chronic medical comorbidities, aspects of care received, medication use, and symptom severity (fibromyalgia impact, fatigue, cognitive symptoms, non-restorative sleep, and functioning). Our primary objective was to evaluate whether Sense of Coherence (SOC) operates as both a mediator and an effect modifier in the relationship between trauma-related variables and fibromyalgia symptoms. Secondary objectives were to examine whether commonly used medication classes (e.g., antidepressants, hypnotics, analgesics, opioids) moderate trauma–symptom associations and to explore combined pharmacological and psychological influences on symptom burden. We hypothesized that adversity would relate to higher symptom severity, that SOC would mediate and potentially moderate adversity–outcome associations, and that medication classes would show differential moderation patterns reflecting both pharmacological effects and clinical selection mechanisms.

## 2. Materials and Methods

### 2.1. Design Overview, Setting, and Participants

This cross-sectional study followed the STROBE guidelines. All participants provided written informed consent prior to participation through a signed informed consent form (ICF). The study was conducted in accordance with the ethical principles of the Declaration of Helsinki. The protocol was approved by the Research Ethics Committee of Hospital de Clínicas de Porto Alegre, Brazil (IRB #2023-0210). The recruitment period ran from 1 August 2023, to 30 November 2023.

### 2.2. Recruitment, Inclusion, and Exclusion Criteria

Individuals volunteered after responding to announcements in public spaces or advertisements on websites such as Facebook and Craigslist. These advertisements were shared through the networks of the National Association of Fibromyalgia and Related Diseases (ANFIBRO). A live online session introduced the project and addressed public questions. A message explaining the study’s purpose was shared on social media to invite people to take part. In addition, the study was disseminated through local media outlets, including television, radio, and newspapers, to broaden community reach beyond patient associations and online platforms. Thus, recruitment was not restricted to a single online platform but relied on a multi-channel, public dissemination strategy.

Interested individuals accessed the study through a provided link. After confirming their interest, they received an informed consent form. Upon signing, participants received a questionnaire about sociodemographic data and the American College of Rheumatology (ACR-2016) questionnaire (Wolfe et al., 2016), via the REDCap (Research Electronic Data Capture) platform. The evaluation collected medical history and symptom details to confirm diagnoses. The research team managed recruitment by sending up to three messages per individual. If registration remained incomplete after 10 days, an additional reminder was sent. After participants completed their registration, they did not receive any additional messages.

To participate, individuals had to be at least 18 years old, able to read, and have a confirmed fibromyalgia diagnosis according to the 2016 American College of Rheumatology (Wolfe et al., 2016). The 2016 ACR criteria stipulate that: (1) generalized pain, defined as pain in at least 4 of 5 regions, must be present; (2) symptoms must be persistent for at least 3 months; (3) the Widespread Pain Index (WPI) must be ≥7 with a Symptom Severity Scale (SSS) score ≥ 5, or a WPI of 4–6 with an SSS score ≥ 9; and (4) the diagnosis is valid regardless of other comorbid conditions, and the presence of fibromyalgia does not exclude additional clinically relevant illnesses. Consistent with these criteria, participants needed a combined WPI + SSS score ≥ 13. Participants were excluded if they did not meet the 2016 ACR criteria or did not complete study questionnaires after three follow-up attempts.

### 2.3. Instruments and Assessment

After providing consent, participants completed the questionnaires via REDCap. To minimize misunderstanding, each item included accessible instructions. All instruments were validated for the Brazilian population, ensuring accurate and reliable measurement. The research team was trained to guide the recruitment process and instruct participants on how to meet the study’s requirements. Response monitoring was conducted continuously, and in cases of incomplete answers, up to three follow-up contacts were made to ensure correction. Weekly meetings were held to address any doubts that arose during the study, enabling evaluators to calibrate. Instructions for all procedures and assessment methods were included in a standardized instruction manual.

### 2.4. Primary Outcome Measures (Dependent Variables)

The Fibromyalgia Impact Questionnaire (FIQ) was used to assess the impact of fibromyalgia on function, symptoms, and overall quality of life across 10 domains, with higher scores indicating greater disease burden (e.g., physical functioning, pain intensity, fatigue, stiffness, sleep quality, mood, work impairment, and ability to perform daily activities), with higher scores indicating greater disease burden. We used the version validated for the Brazilian population (Marques et al., 2006).

The Symptom Severity Scale (SSS) from the 2016 revision of the American College of Rheumatology (ACR) fibromyalgia diagnostic criteria was used to assess the severity of three core somatic symptom domains: fatigue, waking unrefreshed, and cognitive symptoms. Each symptom was rated according to its severity over the past week on a 4-point scale ranging from 0 (no problem) to 3 (severe, pervasive, continuous, or life-disturbing problems) (Wolfe et al., 2016).

#### 2.4.1. Adversity and Psychosocial Measures (Primary Predictors)

Adversity was operationalized using both specific event indicators and cumulative indices reflecting overall trauma burden. Trauma-related variables were assessed using dichotomous items (yes/no) evaluating whether participants associated the onset or worsening of fibromyalgia symptoms with specific adverse experiences, including:

(i) major life events (e.g., loss of a loved one, marriage, job loss);
(ii) verbal aggression causing ongoing distress;
(iii) emotional aggression causing ongoing distress; and
(iv) sexual abuse, including unwanted physical contact, coercive advances, verbal harassment, emotional pressure, or non-consensual sexual acts.

A cumulative adversity index (cumulative abuse; range 0–4) was derived by summing exposure across abuse-related domains, providing an overall measure of trauma burden. In addition, an appraisal-related adversity variable captured self-reported perception that fibromyalgia symptoms were related to persistent physical aggression, reflecting subjective attribution of symptom onset or worsening to aggressive experiences. Thus, adversity was represented in the analyses by both the cumulative abuse index and by specific adversity indicators, allowing evaluation of global trauma load as well as domain-specific effects.

For descriptive analyses, cumulative abuse was additionally categorized into three groups (0, 1, and ≥2 types of abuse) to characterize exposure profiles in Table 1 and Table 2; however, to minimize information loss and reduce misclassification inherent to coarse categorizations, all regression, mediation, and moderation models used the continuous cumulative abuse score (range 0–4) to preserve dose–response information and maximize statistical power.

#### 2.4.2. Psychosocial and Central Pain Processing Measures

Psychological and pain-processing constructs relevant to nociplastic mechanisms were assessed using validated instruments. Pain catastrophizing was measured using the Pain Catastrophizing Scale (PCS; total score 0–52), covering rumination, magnification, and helplessness domains (Sehn et al., 2012). Central sensitization symptoms were assessed using the Central Sensitization Inventory (CSI; 0–100), capturing diffuse pain, fatigue, non-restorative sleep, cognitive difficulties, headaches, and urological complaints (Caumo et al., 2013). Depressive symptoms were evaluated using the Patient Health Questionnaire-9 (PHQ-9; 0–27), with scores ≥ 9 indicating clinically relevant depression in Brazilian populations (Santos et al., 2013). Psychiatric diagnosis burden was defined as non-depressive psychiatric comorbidity and operationalized as a cumulative count of self-reported lifetime diagnoses of anxiety disorders, bipolar disorder, panic disorder, and post-traumatic stress disorder (PTSD); this variable was analyzed separately from depressive symptoms, which were assessed exclusively using the PHQ-9 total score. Kinesiophobia was measured using the Tampa Scale for Kinesiophobia (TSK), reflecting fear of movement and perceived threat related to physical activity (Siqueira et al., 2007). Alcohol consumption patterns were screened using the Alcohol Use Disorders Identification Test—Consumption (AUDIT-C) (Corradi-Webster et al., 2005).

#### 2.4.3. Sociodemographic and Clinical Characteristics

Demographic and clinical covariates included: age (years), educational attainment (years or level of formal schooling), body mass index category, occupational status (unemployed, employed/student/self-employed, retired, disability benefits), and fibromyalgia duration (years) and history of medical comorbidities.

### 2.5. Sense of Coherence (SOC) Composite and Indicator Variables

A dedicated SOC questionnaire was not administered. This choice reflects a deliberate methodological decision aimed at preserving feasibility and data quality in a large online survey and at avoiding excessive respondent burden and overlap with symptom measures. Moreover, Brazilian Portuguese versions of the SOC scale have shown context-dependent psychometric limitations in prior studies (Scalco et al., 2020). Accordingly, SOC was operationalized using a theory-driven composite based on available indicators, an approach intended to be exploratory and hypothesis-generating rather than a substitute for validated SOC instruments. Importantly, these indicators were not intended to represent direct measurements of Antonovsky’s SOC construct, but rather to capture an integrated psychosocial vulnerability/coherence profile inspired by this framework. The PHQ-9 was included as a proxy indicator of affective distress and reduced engagement with life, which are conceptually related to the “meaningfulness” dimension, rather than as a measure of SOC itself. We acknowledge that depression is a distinct clinical construct and a frequent comorbidity in fibromyalgia, and that this choice represents a pragmatic and imperfect operationalization with potential overlapping with clinical outcomes. Accordingly, this composite should be interpreted as a hypothesis-generating, theory-informed proxy rather than as a replacement for validated SOC instruments.

Instead, we constructed a theory-driven SOC surrogate aligned with Antonovsky’s salutogenic framework, operationalizing three theoretical domains: Meaningfulness, Manageability, and Comprehensibility. This SOC surrogate represents an integrated psychosocial functioning profile rather than a stable personality trait, and therefore mediation effects should be interpreted as statistical decomposition of shared psychosocial vulnerability rather than causal psychological pathways. Accordingly, indicators included in each domain were not intended to represent symptom severity per se, but rather perceived bodily threat, predictability, and coherence of illness experience, as well as perceived coping resources and engagement with care, consistent with the theoretical structure of the SOC framework.

The meaningfulness domain was constructed from affective and cognitive-emotional burden indicators, including pain catastrophizing (total PCS score), depressive symptom severity assessed exclusively by the PHQ-9 total score, and psychiatric diagnosis burden reflecting non-depressive psychiatric comorbidity, operationalized as a cumulative count of self-reported lifetime diagnoses of anxiety disorders, bipolar disorder, panic disorder, and post-traumatic stress disorder (PTSD). These variables represent affective and cognitive valuation of symptoms and life context, capturing the extent to which individuals perceive their experiences as emotionally meaningful and interpretable.

The Manageability domain incorporated indicators of functional coping resources, behavioral engagement, and healthcare-related support. Specifically, the following items/variables were included: (i) work status (categorical variable: unemployed, employed, student, caregiver, self-employed, retired, receiving disability benefits); (ii) physical activity (regular participation in physical activities: yes/no); (iii) medication-adherence behaviors, including consistent medication use as prescribed (yes/no), adherence to the recommended treatment plan (yes/no), frequent medication forgetfulness (yes/no; reverse-coded), and self-initiated medication interruption (yes/no; reverse-coded); (iv) treatment engagement and perceived control, including collaborative treatment decision-making (yes/no), patient input on prescribed treatment (yes/no), waiting time for fibromyalgia specialist consultation (ordinal categories), and global treatment quality assessment (Likert-type scale); and (v) complexity of pharmacological treatment (number of medication classes in use). These variables represent internal and external coping resources and, taken together, capture perceived control over health-related demands.

The Comprehensibility domain reflected cognitive understanding and perceived predictability of symptoms and incorporated indicators related to bodily threat appraisal and illness interpretation. Specifically, the following items/variables were included: (i) diagnosis of other pain conditions (musculoskeletal pain: yes/no; neuropathic pain: yes/no); (ii) Central Sensitization Inventory (CSI total score); (iii) Tampa Scale of Kinesiophobia (TSK total score); (iv) perceived linkage between symptoms and physical aggression (yes/no); and (v) age at fibromyalgia onset (categorized as <18 years, 19–25 years, 26–40 years, and >40 years). Although CSI and TSK are commonly used as symptom-related instruments, in this composite they were conceptualized as proxies of bodily threat appraisal and illness predictability rather than as outcome measures, consistent with the theoretical structure of the comprehensibility component of the SOC framework. Together, these indicators capture perceived threat, coherence, and interpretability of bodily experiences.

All indicators were directionally aligned so that higher values consistently reflected stronger SOC, and subsequently standardized (z-scores) to ensure comparability across scales. Domain-specific SOC scores were calculated as the means of standardized indicators within each domain. A global SOC composite was computed as the mean of the three domain scores and then standardized (SOC_new_composite_z) for use in all regression, mediation, and moderation analyses. A detailed flowchart of the data transformation process and the full list of variables included in each domain are provided in Supplementary Figure S1.

**Figure 1:**
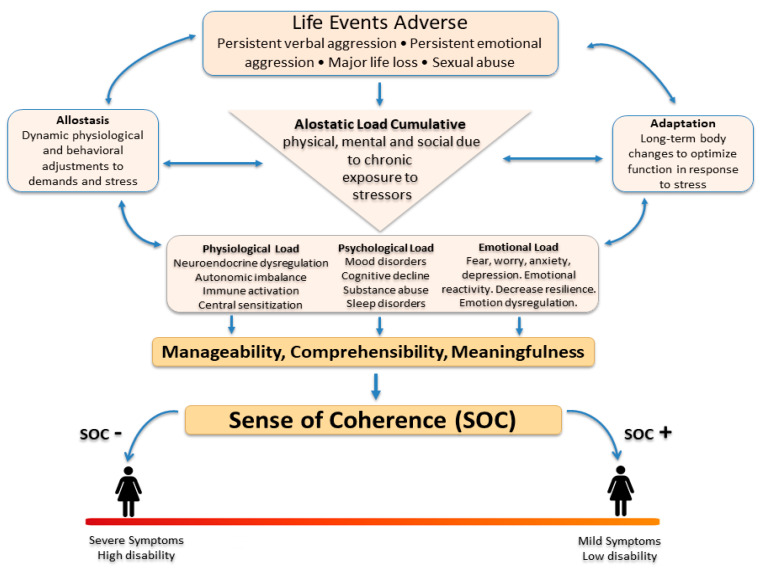
Biopsychosocial model of fibromyalgia severity and the modulating role of sense of coherence (SOC). Adverse life events increase emotional, psychological, and physiological load, leading to greater symptom severity through allostatic dysregulation. SOC influences this process by mediating the impact of adversity and buffering its effects on functional impairment. Lower SOC is associated with higher symptom burden and greater vulnerability to the effects of cumulative stress, representing a key mechanism linking adverse experiences to fibromyalgia severity. Unidirectional arrows represent hypothesized directional relationships between variables, whereas bidirectional arrows indicate reciprocal or correlational associations within the conceptual model.

Figure 1 summarizes the conceptual framework underlying the SOC composite and its role within the biopsychosocial pathways linking adverse experiences to fibromyalgia severity. The model integrates SOC dimensions with cumulative biological and psychosocial stress (allostatic load), illustrating how lower SOC amplifies symptom burden and functional impairment.

### 2.6. Covariates Included in Adjusted Models

All adjusted regression, mediation, and moderation models controlled for age, educational attainment, body mass index category and disability due to pain in daily activities.

### 2.7. Medication Constructs

For exploratory moderation analyses, we considered several medication groups as potential effect modifiers: (i) Antidepressants: tricyclic antidepressants, dual-action antidepressants, a combined tricyclic/dual indicator, and SSRIs (selective serotonin reuptake inhibitors). (ii) Sleep-related medications: melatonin, zolpidem, and benzodiazepines. (iii) Analgesic variables: non-opioid analgesics (NSAIDs, dyclonine/dyprinon, acetaminophen), use opioid (any opioid use), and specific of opioid agents (codeine, morphine, oxycodone, methadone, and tramadol). These medication-related moderation analyses were prespecified as exploratory and secondary, aimed at contextualizing the primary SOC-based models rather than redefining the main mechanistic findings.

### 2.8. Efforts to Address Potential Sources of Bias

To minimize potential sources of bias, all study procedures were fully standardized through REDCap, ensuring identical administration, automated data capture, and removal of duplicate entries. The platform was configured to prevent repeated submissions, and suspicious or duplicate entries were screened and excluded prior to analysis. Recruitment was broad and public, and all instruments used were validated for the Brazilian population, reducing measurement error. Sensitive items (e.g., trauma exposures) were self-administered online to limit bias to social desirability. The research team followed a structured operations manual, with weekly calibration meetings and continuous data-quality monitoring. Incomplete responses triggered up to three automated reminders to reduce attrition bias. In addition, only participants meeting the 2016 ACR fibromyalgia criteria after full questionnaire completion were included in the final analytical sample, providing an additional clinical consistency check on eligibility.

For the construction of the SOC composite, indicators were theory-driven and grouped into meaningfulness, manageability, and comprehensibility domains. All indicators were standardized, and reverse-coded, when necessary, to maintain consistent directionality. By selecting conceptually distinct and non-redundant indicators, we minimized collinearity within SOC domains and avoided inflation of mediation or moderation effects. All regression models were adjusted for key demographic and clinical confounders (age, education, BMI, disability due to pain in daily activities) to reduce residual confounding.

Missing data was minimized through structured follow-up to reduce incomplete questionnaires. SOC domain scores were calculated from the means of available indicators, preserving the construct’s theoretical structure and avoiding listwise deletion at the domain-construction stage. For inferential analyses, complete-case datasets were used, and all regression models were estimated to use the same analytic sample, as reflected by the uniform sample size reported in the tables. This approach ensures comparability across models and outcomes while avoiding biases introduced by data imputation.

### 2.9. Statistical Analysis

Descriptive analyses were conducted by categorizing participants according to cumulative abuse exposure (no abuse, one type of abuse, and two or more types of abuse), as presented in Table 1 and Table 2. Baseline characteristics were summarized across these groups to examine differences in sociodemographic, clinical, and psychosocial profiles. Categorical variables were expressed as frequencies and percentages, and continuous variables as means and standard deviations. Group comparisons were performed using χ^2^ tests for categorical variables and independent-sample t tests or Mann–Whitney U tests for continuous variables, as appropriate. For descriptive purposes, cumulative abuse was categorized into three clinically interpretable groups; however, all inferential analyses used the continuous cumulative abuse score (range 0–4) to preserve dose–response information and maximize statistical power. Because cumulative abuse categories were derived from the same adversity indicators presented in descriptive tables, these variables are not independent across groups and should be interpreted as characterization of exposure profiles rather than as independent between-group comparisons.

All inferential analyses were conducted using linear regression models, with cumulative abuse (range 0–4) as the main adversity predictor and fibromyalgia impact (FIQ total score) as the primary outcome. Secondary outcomes included fatigue, cognitive symptoms, and non-restorative sleep, as measured by the Symptom Severity Scale. Unadjusted associations were first examined using simple linear regression (Outcome = β_0_ + β_1_ × Cumulative_abuse + ε). Adjusted models were then estimated including age (years), educational attainment (years of schooling), body mass index (BMI category), and disability due to pain in daily activities, excluding SOC from the covariate set. All mediation and moderation models were additionally adjusted for the same covariates.

To evaluate whether sense of coherence (SOC_new_composite_z) mediated the associations between cumulative abuse and each clinical outcome, regression-based mediation models were specified using a path-analytic framework: SOC_new_composite_z = α_0_ + α_1_ × Cumulative_abuse + ε_a_ (path a), and Outcome = γ_0_ + γ_1_ × Cumulative_abuse + γ_2_ × SOC_new_composite_z + ε_γ_ (paths b and c′). Indirect effects were calculated as the product a × b and tested using Sobel tests and were additionally confirmed using nonparametric bootstrapped confidence intervals based on 5000 resamples of the original dataset. Mediation analyses were conducted separately for each outcome and for each adversity indicator (cumulative abuse and specific adversity domains).

To test whether SOC modified the association between cumulative abuse and clinical outcomes, interaction models were estimated as Outcome = δ_0_ + δ_1_ × Cumulative_abuse + δ_2_ × SOC_new_composite_z + δ_3_ × (Cumulative_abuse × SOC_new_composite_z) + ε. A statistically significant interaction coefficient (δ_3_) was interpreted as evidence that SOC modifies (buffers or amplifies) the effect of adversity on symptom severity. Exploratory moderation analyses were also conducted to examine whether medication classes and selected clinical variables modified adversity–outcome associations, using the same interaction framework.

Missing data were minimized through structured follow-up. SOC domain scores were calculated from the means of available indicators to preserve the construct’s theoretical structure and avoid listwise deletion at the domain-construction stage. For inferential analyses, complete-case datasets were used, and all regression models were estimated using the same analytic sample, as reflected by the uniform sample size reported in the tables. For all models, regression coefficients, 95% confidence intervals, and *p* values were reported. Model assumptions (linearity, homoscedasticity, and normality of residuals) were assessed using standard diagnostic plots. All statistical analyses were performed using IBM SPSS Statistics for Windows, version 22 (IBM Corp., Armonk, NY, USA), and R software (version 4.4.2; R Foundation for Statistical Computing, Vienna, Austria). Statistical significance was set at *p* < 0.05 (two-tailed).

## 3. Results

### 3.1. Sample Baseline Characteristics

From the publicly available online survey, 4200 individuals initiated the questionnaire. After removal of duplicate entries, 3500 unique respondents remained, of whom 2495 (71.3%) completed all required instruments. After excluding 295 respondents who did not meet fibromyalgia screening criteria and 101 men due to insufficient sample size for sex-stratified analyses, the final analytical cohort comprised 2099 women.

Table 1 summarizes the demographic, socioeconomic, clinical, medication-use, and healthcare engagement characteristics of the study sample. Participants exposed to higher levels of cumulative abuse presented a more vulnerable social and clinical profile, including higher rates of work disability, greater use of psychotropic and pain-related medications, and indicators of lower treatment adherence and access to specialized care. These differences characterize a population with greater clinical complexity and justify adjustment for age, body mass index, and work disability in subsequent regression models.

### 3.2. Fibromyalgia Severity and Psychological Burden

Table 2 presents symptom severity scores according to cumulative abuse exposure categories (no abuse, one type of abuse, and two or more types of abuse). A graded increase in symptom burden was observed across abuse categories, particularly for fibromyalgia impact, cognitive symptoms, and non-restorative sleep, indicating a dose–response pattern between adversity exposure and clinical severity. Differences in fatigue scores across groups were comparatively smaller.

### 3.3. Validation and Distribution of SOC Surrogate

Discriminant validity was evaluated using correlation analyses between SOC domains and clinical outcomes (Supplementary Table S2). Correlations were of low to moderate magnitude, and no coefficients approached unity, indicating that SOC constructs are related to, but not redundant with, symptom severity measures. These findings support the conceptual distinctiveness of the SOC surrogate in relation to clinical outcomes, suggesting that it captures psychosocial organization of coping and illness interpretation rather than simply reflecting symptom burden.

### 3.4. Association Between Cumulative Abuse and Clinical Outcomes

Table 3 summarizes adjusted linear regression models examining the association between cumulative abuse exposure (range 0–4) and symptom severity across four clinical domains: fibromyalgia impact (FIQ), cognitive dysfunction, fatigue, and non-restorative sleep. After adjustment for age, body mass index (BMI), and disability due to pain in daily activities, cumulative abuse was significantly associated with higher symptom burden across all outcomes. The positive linear coefficients indicate a dose–response relationship between increasing levels of lifetime adversity and multidimensional clinical severity.

Supplementary Table S3 shows analogous models using individual adversity indicators, with generally weaker and less consistent associations across symptom domains compared with cumulative abuse.

### 3.5. Sense of Coherence and Symptom Outcomes

#### 3.5.1. Mediation Analysis: SOC as a Pathway Linking Adversity to Symptom Severity

Mediation models were estimated to test whether SOC_new_composite_z mediated the associations between adversity indicators and each clinical outcome.

**Figure 2:**
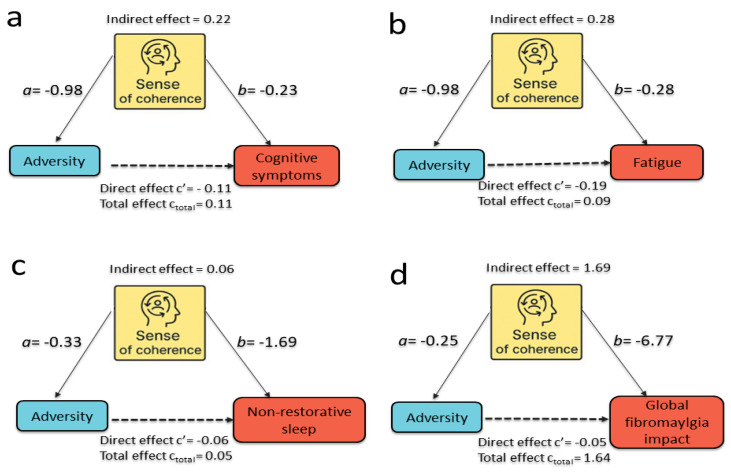
SOC Mediates the Impact of Adversity on Cognitive, Fatigue, Sleep, and Global Symptom Burden. Mediation models illustrating how sense of coherence (SOC) statistically transmits the association between adversity and four fibromyalgia symptom domains (**a**–**d**). Across all models, higher adversity was associated with lower SOC (path a), and lower SOC with worse outcomes (path b), yielding positive indirect effects (a × b) that were larger than or comparable to the remaining direct effects (c′). (**a**) Cognitive symptoms: All adversity indicators showed strong mediated effects. For persistent physical aggression, adversity predicted lower SOC (a = −0.98) and higher SOC predicted fewer cognitive symptoms (b = −0.23). The indirect effect (a × b = 0.22) exceeded the total effect (c_total = 0.11), and the adjusted direct effect was small and negative (c′ = −0.11). Similar patterns were observed for loss, emotional and verbal aggression, sexual abuse, and cumulative abuse. (**b**) Fatigue: SOC strongly mediated the adversity–fatigue association. For persistent physical aggression, a = −0.98 and b = −0.28; the indirect effect (a × b = 0.28) exceeded the total effect (c_total = 0.09), and the direct effect became negative after adjustment (c′ = −0.19). (**c**) Non-restorative sleep: Adversity indicators were associated with lower SOC, and lower SOC with more severe non-restorative sleep. Indirect effects were smaller than for cognitive symptoms and fatigue but remained consistent and statistically meaningful. (**d**) Global fibromyalgia impact (FIQ): For cumulative abuse, adversity predicted lower SOC (a = −0.25) and higher SOC predicted lower FIQ (b = −6.77). The indirect effect (a × b = 1.69) approximated the total effect (c_total = 1.64), and the direct effect was minimal and slightly negative (c′ = −0.05), a pattern also observed for other adversity indicators. Solid arrows represent the direct paths tested in the mediation model. Dashed arrows represent moderating effects (interaction terms) included in the analysis.

Figure 2a–d presents the mediation analyses assessing whether SOC_new_composite_z mediated the associations between multiple adversity indicators and the four clinical domains: symptoms of cognitive dysfunction, fatigue, non-restorative sleep, and global fibromyalgia impact (FIQ). Each adversity variable (including cumulative abuse, physical, verbal, emotional aggression, loss events, sexual abuse and the appraisal variable (self-reported perception that fibromyalgia symptoms are related to persistent physical aggression) was entered separately as the predictor, SOC_new_composite_z as the mediator, and one symptom domain as the outcome. Across all mediation models, the pattern was consistent and theoretically coherent. Higher adversity was associated with lower SOC (negative a-paths), In turn, higher SOC was associated with lower symptom severity, reflected in negative b-paths. Consequently, indirect effects (the product a × b) were uniformly positive, indicating that adversity contributes to symptom burden in part through reductions in SOC.

In several models, the estimated indirect effect of adversity on outcomes via SOC was larger than the corresponding total effect. This pattern reflects inconsistent mediation (suppression), in which the indirect effect (a × b) and the direct effect (c′) have opposite signs. In practical terms, this indicates that the pathway through SOC captures a strong association between adversity and symptom severity, while the residual direct association operates in the opposite direction, partially offsetting the total effect. This phenomenon does not indicate a model error but rather a statistical suppression pattern. Multicollinearity was examined and excluded as an explanation (all VIFs < 2.0), and the indirect effects were confirmed using bootstrapped confidence intervals. Accordingly, these findings should be interpreted as statistical decomposition of cross-sectional associations rather than as evidence of a simple causal mediation process.

#### 3.5.2. Mediation Findings Across Clinical Domains

Table 4 provides full mediation models for all adversity indicators across cognitive symptoms, fatigue, non-restorative sleep, and global fibromyalgia impact (FIQ). For comparability across predictors, paths “a” and “b” are reported as standardized coefficients (β). Adversity exposures consistently predicted lower SOC (negative a-paths), and lower SOC was strongly associated with greater symptom severity (negative b-paths). In many cases, indirect effects exceeded direct effects and, at times, surpassed the magnitude of the total effect, indicating predominant or near-complete mediation. These effects were strongest for fatigue and cognitive dysfunction. Notably, several proportion mediated values exceed 1, reflecting a statistical suppression (inconsistent mediation) pattern, in which the indirect effect (a × b) and the direct effect (c′) have opposite signs. In this situation, the proportion mediated can exceed 1 and should not be interpreted as a calculation error, but rather as an indication that the mediated and direct pathways operate in opposing directions.

Overall, SOC emerges as a central regulatory mechanism linking adverse experiences to clinical symptomatology, explaining a meaningful proportion of symptom variability in fibromyalgia, and reinforcing the importance of SOC as a target for both mechanistic understanding and potential intervention strategies.

### 3.6. Moderation Analyses: SOC and Clinical Factors as Modifiers of Adversity Effects

This section examines whether the associations between adverse experiences and clinical outcomes vary according to individual and clinical contextual factors. First, we evaluated whether sense of coherence (SOC) modifies the strength of adversity–symptom relationships. Subsequently, we explored whether medication use and selected clinical variables act as additional moderators of these associations. SOC-based moderation models were considered the primary analyses, whereas medication and comorbidity interactions were treated as secondary and exploratory.

#### 3.6.1. Moderation by Sense of Coherence (SOC)

To determine whether SOC also modified (moderated) the strength of adversity–symptom associations, we estimated models including *adversity × SOC_new_composite_z interaction terms*. For symptoms of cognitive dysfunction and non-restorative sleep, *none of the adversity × SOC interactions* reached conventional statistical significance, indicating that SOC influences these domains primarily as a mediating pathway rather than as a moderator of adversity effects.

In contrast, SOC significantly moderates the impact of adversity on fatigue. Positive interaction coefficients were observed for loss (β = 0.052, *p* = 0.009), verbal aggression (β = 0.060, *p* = 0.004), emotional aggression (β = 0.057, *p* = 0.005), sexual abuse (β = 0.057, *p* = 0.027), two abuses; β = 0.024, *p* = 0.001), and three or more abuses (β = 0.026, *p* = 0.028). These positive interaction terms indicate that the association between adversity and fatigue varies across SOC levels. Individuals with lower SOC exhibited stronger adversity–fatigue associations, whereas individuals with higher SOC showed attenuated associations. In the context of the strong negative main effect of SOC on fatigue, this pattern reflects a dual role for SOC, functioning both as a mediator and as a buffer of adversity-related fatigue.

**Figure 3:**
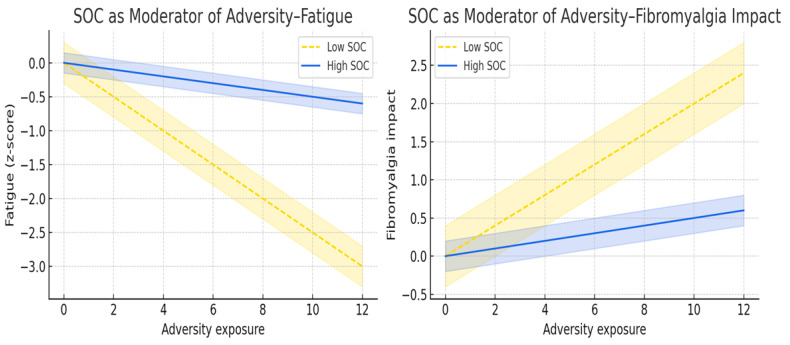
SOC as a Moderator of the Association between Adversity Exposure and Clinical Outcomes. The plots show the moderating role of sense of coherence (SOC) on the association between cumulative adversity and two fibromyalgia outcomes: fatigue (**left**) and global impact (FIQ; **right**). Predicted values are displayed for low SOC (dashed yellow) and high SOC (solid blue), with 95% confidence bands. Steeper slopes in the low-SOC group indicate stronger adversity–symptom associations, consistent with attenuation of clinical severity at higher SOC levels. The *x*-axis shows cumulative adversity, and the *y*-axes show standardized symptom severity (z-scores). Negative z-scores in the fatigue panel indicate greater fatigue, whereas positive z-scores in the FIQ panel indicate worse global impact. Opposite slope directions reflect domain-specific effects across outcomes. The shading in the figure represents the 95% confidence interval (CI).

A similar moderating pattern emerged for fibromyalgia impact. SOC significantly modified the associations between FIQ and verbal aggression (β = 1.58, *p* = 0.002), emotional aggression (β = 1.44, *p* = 0.004), sexual abuse (β = 2.11, *p* = 0.001), two abuses (β = 0.75, *p* < 0.001), and three or more abuses (β = 0.57, *p* = 0.039). As with fatigue, positive interaction terms, combined with the strong protective main effects of SOC on FIQ, indicate that SOC shapes the degree to which adversity is expressed in global fibromyalgia impact. Higher SOC diminishes the translation of adversity into disability and symptom burden, demonstrating that SOC functions not only as a pathway (mediator) but also as a contextual factor (moderator) that alters the magnitude of adversity-related clinical severity. Together, Figure 3 demonstrates that SOC not only mediates but also moderates the clinical expression of adversity, particularly for fatigue and global impact, highlighting SOC as a multidimensional regulatory factor in fibromyalgia.

Overall, the moderation models shown in Table 5 reveal a clear and domain-specific pattern: SOC did not moderate the relationship between adversity and cognitive symptoms or non-restorative sleep but consistently modified the effects of adversity on fatigue and global fibromyalgia impact (FIQ). The significant positive interaction terms for multiple adversity indicators in these two domains indicate that individuals with lower SOC exhibit steeper adversity–symptom slopes, whereas those with higher SOC show attenuated associations. These findings suggest that SOC operates as a relative buffering factor for fatigue and global impact, although the magnitude of the interactions indicates attenuation rather than large clinically transformative effects.

#### 3.6.2. Medication Use and Clinical Factors as Moderators

Given that this composite includes domains closely related to symptom severity and emotional distress, we acknowledge the risk of construct overlap with the outcomes, particularly in mediation models. Therefore, these analyses should be interpreted as exploratory and descriptive rather than as evidence of a distinct causal psychological mechanism. In line with this exploratory and descriptive framework, we also examined whether commonly used medication classes modified these associations.

Regression models with interaction terms for adversity × medication were estimated for cognitive symptoms, FIQ, and non-restorative sleep. Medications included antidepressants (tricyclics, dual-action agents, combined tricyclic plus dual agents, and SSRIs), benzodiazepines, sleep agents (melatonin and zolpidem), non-opioid analgesics, and opioids (codeine, morphine, oxycodone, methadone, and tramadol).

### 3.7. Comorbidities and Medication Use as Moderators and Mediators of Adversity–Outcome Associations

Moderation effects were most evident for cognitive symptoms. Dual-action antidepressants amplified the association between persistent physical aggression and cognitive dysfunction (β = 0.21, *p* = 0.002), with a smaller effect for combined dual-plus-tricyclic therapy (β = 0.12, *p* = 0.023). Two types of abuse also interacted positively with dual-action antidepressants (β = 0.05, *p* = 0.034). No consistent medication-moderation effects emerged for non-restorative sleep. Zolpidem showed a potential buffering effect on global fibromyalgia impact, with a negative interaction with persistent physical aggression (β = −4.55, *p* = 0.041).

Overall, dual-action antidepressants amplified adversity–cognition associations, whereas zolpidem attenuated adversity–FIQ effects; both patterns remain exploratory and require replication.

Supplementary Table S4A shows the moderation and mediation analyses examining whether clinical comorbidities and medication use modify or partially explain the associations between adverse experiences and symptom severity. Moderation models assessed interaction effects involving scheduled consultations, stroke, and several medication classes—including antidepressants, benzodiazepines, sleep agents, non-opioid analgesics, and opioids—across cognitive dysfunction, fatigue, non-restorative sleep, and global fibromyalgia impact (FIQ). Significant interactions were observed for specific medication classes and clinical factors, indicating that these variables can alter the strength or direction of adversity–symptom relationships.

Supplementary Table S4B shows the mediation analyses further evaluated whether hypertension, diabetes, and related comorbidities served as intermediary pathways linking adversity to clinical outcomes. While small but statistically significant indirect effects were detected, their magnitude was modest compared to psychosocial mechanisms, reinforcing the limited explanatory contribution of comorbidities and medication use to the adversity–symptom pathways.

## 4. Discussion

### 4.1. Main Findings

This study examined associations among cumulative adversity, sense of coherence (SOC), and clinical severity in individuals with fibromyalgia within a cross-sectional observational framework. Greater exposure to adverse experiences was consistently associated with higher symptom burden across multiple domains, including fibromyalgia impact (FIQ), fatigue, cognitive symptoms, and non-restorative sleep. Lower SOC was associated with worse outcomes, and SOC statistically accounted for a substantial proportion of the observed adversity–symptom associations. In addition, SOC modified the strength of adversity-related effects for selected outcomes, particularly fatigue and global disease impact. All findings should be interpreted as associations, not as evidence of causal directionality or biological mechanisms. Although multiple interaction models were tested, the central and most consistent finding of the study is the mediating and moderating role of SOC; medication- and comorbidity-related interactions showed smaller, less consistent, and exploratory effects.

### 4.2. Construct Validity of the SOC Surrogate

SOC was conceptualized according to Antonovsky’s salutogenic framework (Antonovsky, 1987; Kaleycheva et al., 2021) and operationalized using a theory-driven composite integrating emotional appraisal, coping resources, engagement with care, and cognitive interpretation of bodily symptoms. Indicators included depressive symptoms, pain catastrophizing, fatigue, central sensitization, kinesiophobia, psychiatric comorbidity burden, functional status, physical activity, and treatment engagement. This composite was designed to capture the core psychosocial construct of interest and constitutes the central analytical focus of the study, whereas additional clinical and treatment-related interactions were examined as secondary and exploratory layers of analysis. Because several of these indicators are intrinsically related to symptom experience in fibromyalgia, potential overlap with outcome measures represents an important concern. However, correlation analyses showed only moderate associations between SOC domains and clinical outcomes, suggesting that the constructs are not redundant. In addition, exploratory latent structure analyses supported separable latent dimensions for SOC and symptom severity. These findings are consistent with prior work showing that resilience-related constructs capture broader psychosocial vulnerability beyond symptom intensity alone (Eriksson & Lindström, 2006; Iannuccelli et al., 2025). For this reason, potential circularity should be considered a central limitation of the present mediation models, rather than a secondary methodological concern, as part of the observed indirect effects may reflect shared construct variance between the SOC surrogate and symptom outcomes rather than independent pathways. Nevertheless, SOC in this study cannot be interpreted as an entirely independent psychological trait, as it incorporates dimensions closely linked to emotional distress and functional impairment. Rather, it reflects an integrated psychosocial profile related to how individuals interpret, cope with, and manage chronic illness.

### 4.3. SOC as a Psychosocial Pathway Linking Adversity and Symptoms

Our mediation analyses indicate that sense of coherence (SOC) accounts for a substantial proportion of the association between cumulative adversity and symptom severity, particularly for fatigue and cognitive dysfunction. This pattern should be interpreted as a statistical decomposition of cross-sectional associations rather than evidence of a causal or biological pathway. In this context, SOC likely represents an integrated psychosocial vulnerability profile that brings together emotional distress, coping resources, functional engagement, and illness appraisal, in line with contemporary biopsychosocial and stress-regulation frameworks (Yoo & Kim, 2024; Giglio et al., 2025). Within fibromyalgia—a prototypical nociplastic pain condition—this profile may help explain why similar levels of adversity translate into different degrees of symptom burden, without implying specific biological mechanisms.

This interpretation is consistent with, and strengthened by, recent biopsychosocial frameworks that place meaning-making, emotional support, and relational contexts at the center of chronic disease adaptation. For instance, La Rosa and Commodari (2025) describe how psychosocial resources and emotional support needs across the lifespan shape symptom experience and adjustment in women with chronic pain conditions, highlighting the role of illness meaning-making and relational support in long-term adaptation. In a related vein (La Rosa & Commodari, 2025), Ferrarese et al. (2025) show that resilience-related processes in inflammatory bowel disease are closely linked to anxiety and depression, illustrating how psychosocial resources modulate emotional burden and symptom expression in chronic illness (Ferrarese et al., 2025). Together, these perspectives provide an external conceptual anchor for our findings, supporting the view that SOC reflects a broader organization of coping and meaning-making rather than a single psychological dimension.

Against this conceptual background, it is important to emphasize the limits of our empirical measures. Although quantitative sensory testing was not performed in the present study, and indicators related to central sensitization, movement-related fear, and rumination do not provide direct evidence of altered nociceptive processing, these measures capture dimensions commonly associated with nociplastic pain mechanisms in fibromyalgia and related conditions (Giglio et al., 2025; Goubert & Trompetter, 2017). Nevertheless, reverse and bidirectional relationships remain plausible: persistent symptoms and disability may further erode perceived coherence and coping capacity, reinforcing maladaptive cycles. Accordingly, any reference to nociplastic mechanisms or biological embedding should be understood strictly within a cross-sectional, conceptual framework rather than as empirical confirmation. In this regard, although we did not assess biological markers such as HPA axis function, inflammatory mediators, or neuroendocrine measures, recent evidence has linked fibromyalgia and other nociplastic pain conditions to autonomic dysfunction, HPA axis dysregulation, low-grade systemic inflammation, and alterations in stress-processing neural networks (Yoo & Kim, 2024). Because such markers were not measured here, our results do not demonstrate biological embedding per se; rather, they indicate that SOC captures an integrated psychosocial vulnerability phenotype linking adversity exposure to symptom expression, consistent with broader biopsychosocial and allostatic load frameworks of chronic pain. In this broader perspective, our findings suggest that SOC functions as an integrative psychosocial pathway through which adversity and symptom burden are statistically linked, consistent with contemporary biopsychosocial models of chronic pain.

### 4.4. SOC as a Modifier of Vulnerability

Consistent with its role as a psychosocial regulatory factor, SOC also emerged as a modifier of vulnerability, attenuating the associations between cumulative adversity and selected symptom domains, particularly fatigue and global fibromyalgia impact. This moderation pattern suggests differential susceptibility rather than direct physiological buffering and aligns with contemporary resilience and self-regulation frameworks (Eccleston & Crombez, 2017; Martinez-Calderon et al., 2021). In current stress–pain models, psychosocial resources such as perceived control, coping flexibility, and self-efficacy modulate how stressors are translated into clinical burden, influencing both behavioral responses and physiological stress reactivity (Martinez-Calderon et al., 2021). In this broader framework, these findings also help situate fibromyalgia within a broader spectrum of women’s health conditions characterized by chronic symptoms, emotional burden, and complex biopsychosocial interactions. A recent systematic review and meta-analysis by Li et al. (2025) in endometriosis—a condition that shares important features with fibromyalgia, including chronic pain, high psychological comorbidity, and substantial functional impact—demonstrates a strong association between chronic gynecological disease and mental health outcomes (Li et al., 2025). This broader perspective reinforces the view that vulnerability and resilience processes observed in fibromyalgia are not unique to this condition but reflect shared biopsychosocial dynamics across women’s chronic health conditions.

The domain-specific nature of the moderation effects observed suggests that SOC may differentially influence symptom clusters through distinct behavioral and psychosocial pathways. Fatigue and global functional impairment are strongly shaped by motivational processes, behavioral activation, activity avoidance, and coping strategies—domains closely linked to the manageability and meaningfulness components of SOC (Eccleston & Crombez, 2017; Nijs et al., 2023). In contrast, cognitive dysfunction and sleep disturbances may be more directly associated with neurobiological and chronobiological dysregulation and therefore less responsive to psychosocial buffering alone (Yoo & Kim, 2024; Jurado-Priego et al., 2024). This heterogeneity supports stratified and mechanism-informed treatment approaches, in which psychosocial interventions may preferentially benefit functional and fatigue-related domains, while other symptom clusters may require complementary biological or chronobiological strategies (Yoo & Kim, 2024; Clauw, 2015).

### 4.5. Interpretation Relative to Biomedical Comorbidities

Clinical comorbidities and medication classes showed modest moderation and mediation effects compared with SOC. These analyses were intentionally designed to be exploratory and hypothesis-generating, and they should not be interpreted as redefining the core SOC-centered model of the study. This is expected, as SOC incorporates psychological distress, functional capacity, and health behavior engagement, which are closely associated with nociplastic pain expression. These findings should not be interpreted as minimizing biomedical contributions, but rather as highlighting that psychosocial processes are tightly interwoven with clinical manifestations of fibromyalgia (La Rosa & Commodari, 2025; Häuser et al., 2015). Although hypertension, diabetes, stroke, and healthcare utilization showed statistically detectable effects in moderation and mediation models, their magnitude was consistently small compared with psychosocial pathways. This pattern suggests that while biomedical comorbidities contribute to clinical complexity, they explain only a limited proportion of symptom variability in high-impact fibromyalgia, particularly when central sensitization and psychosocial dysregulation are prominent. The amplification of adversity–cognition associations among users of dual-action antidepressants likely reflects confounding by indication, as patients with higher emotional distress and cognitive burden are more frequently prescribed these agents. This underscores the importance of interpreting medication-related moderation effects within the clinical context rather than as pharmacological mechanisms per se.

### 4.6. Clinical and Translational Implications

The present findings highlight the relevance of psychosocial dimensions—such as coping strategies, illness understanding, treatment engagement, and physical activity—in the clinical expression of fibromyalgia, in addition to pharmacological management. However, this study does not allow inference regarding treatment efficacy or prevention strategies. Contemporary evidence indicates that fibromyalgia treatment should be multimodal, personalized, and mechanism-informed, integrating psychosocial, physical, and pharmacological strategies according to dominant symptom domains and underlying pathophysiological processes (Clauw, 2015). In this framework, behavioral and educational interventions are particularly relevant for improving functional capacity and fatigue, whereas cognitive dysfunction and sleep disturbances may require complementary neurobiological and chronobiological approaches. Accordingly, multimodal programs including pain neuroscience education, graded exercise, behavioral activation, and shared decision-making are consistent with approaches that aim to enhance manageability and comprehensibility, thereby reducing the clinical impact of adversity and facilitating sustained engagement in rehabilitation (Nijs et al., 2023; Jurado-Priego et al., 2024). SOC-related constructs may also help identify patient subgroups with heightened vulnerability to persistent symptoms, supporting stratified and personalized treatment strategies.

From a translational perspective, these findings support incorporating resilience-oriented constructs into both clinical assessment and intervention design. Screening for SOC-related dimensions—such as illness understanding, perceived controllability, and engagement with care—may help identify patients at higher risk for persistent disability and suboptimal response to pharmacological strategies alone. Interventions explicitly targeting coherence-building processes, including structured pain neuroscience education, acceptance-based therapies, narrative approaches, and goal-oriented rehabilitation programs, represent plausible strategies to support adaptive coping and may be relevant for treatment responsiveness, which should be tested in longitudinal and interventional studies (Eccleston & Crombez, 2017; Martinez-Calderon et al., 2021). Importantly, these approaches may be particularly relevant for patients with high trauma burden, in whom purely biomedical interventions are unlikely to adequately address the multifactorial drivers of symptom persistence.

### 4.7. Limitations

Several limitations must be considered when interpreting these findings. *First*, the SOC measure used in this study was a theory-driven composite rather than a validated Antonovsky SOC instrument and incorporates indicators closely related to symptom experience in fibromyalgia. In particular, the inclusion of the PHQ-9 (depressive symptoms) in the meaningfulness domain represents the main source of potential circularity, as depressive symptomatology is also a core component of fibromyalgia impact and fatigue outcomes. Therefore, partial overlaps between the mediator and the outcomes cannot be excluded and may inflate indirect effects through shared construct variance rather than reflecting independent pathways. For this reason, the SOC surrogate should be interpreted as an integrated psychosocial vulnerability/coherence profile rather than as a distinct psychological mechanism. *Second*, the cross-sectional design of this study precludes any inference about temporal ordering or causal relationships among adversity, Sense of Coherence (SOC), and symptom outcomes. Accordingly, the mediation models should be interpreted as statistical decompositions of cross-sectional associations, rather than as evidence of psychological or biological pathways. Reverse and bidirectional relationships remain plausible, and the observed patterns cannot establish directionality. *Third*, consistent with these measurement and design constraints, the mediation findings should be understood as describing patterns of association within a psychosocial–clinical network, not as demonstrating mechanistic or causal mediation. In this context, the results are best viewed as hypothesis-generating, indicating that adversity, psychosocial vulnerability/coherence, and symptom severity are strongly interrelated, but not establishing the direction or independence of these relationships (Aguilar-Latorre et al., 2023; Scalco et al., 2020). *Fourth*, the cumulative adversity index did not assess severity, timing, or duration of exposure, limiting developmental inferences and preventing evaluation of sensitive periods (Spadoti Dantas et al., 2014). *Fifth*, recruitment through online platforms and patient advocacy networks may have introduced selection bias toward individuals with higher symptom burden and healthcare engagement (Bonanato et al., 2009). Also, the sample consisted exclusively of women with high-impact fibromyalgia and substantial psychiatric comorbidity, which limits generalizability but reflects the clinical population most affected by persistent disability. Finally, future studies using validated SOC instruments, independent outcome measures, and longitudinal or interventional designs are required to test temporal ordering, reduce construct overlap, and determine whether the observed associations persist when circularity is minimized. These steps will be essential to clarify whether SOC, as originally defined, plays an independent role in shaping vulnerability or resilience in fibromyalgia, beyond shared variance with symptom-related constructs.

### 4.8. Conclusions

In this cohort of individuals with fibromyalgia, cumulative adversity was consistently associated with greater symptom burden, and lower sense of coherence was associated with worse clinical outcomes across multiple domains. SOC functioned both as an intermediate psychosocial pathway linking adversity to symptom severity and, for selected outcomes, as a modifier of vulnerability to adversity-related symptom amplification. These findings support the relevance of integrated biopsychosocial frameworks and highlight the importance of psychosocial resilience-related processes in the clinical expression of fibromyalgia.

## Figures and Tables

**Table 1:** Sociodemographic, Clinical and Care-Engagement Characteristics according to cumulative abuse exposure (no abuse, one type of abuse, and two or more types of abuse). Data area presented as mean (SD), median (interquartile) or % (*n* = 2099).

		Cumulative Abuse Exposure			
Variables		No History of Abuse (*n* = 1149)	One Type of Abuse (*n* = 354)	Two or More Types of Abuse (*n* = 596)	*p*-Value
Demographic, Educational, and Occupational Characteristics					
Age (ys)		50.94 (10.50)	49.91 (9.66)	50.69 (10.01)	0.25
Formal education (ys)		10.8 (4.2)	9.6 (4.1)	8.9 (3.80)	<0.001
Alcohol Use Disorders Identification Test Consume (AUDIT-C)		1.38 (1.82)	1.63 (2.10)	1.64 (2.19)	0.01
Race/Ethnicity (Black/Brown) (Yes)		196 (52.4%)	65 (17.4%)	113 (30.2%)	0.59
Occupational status category					
	Unemployed (Yes)	144 (12.5%)	46 (13.0%)	100 (16.8%)	0.04
	Employed/Student/Caregiver/Self-employed (Yes)	630 (54.8%)	195 (55.1%)	306 (51.3%)	0.33
	Retired (Yes)	212 (18.5%)	57 (16.1%)	93 (15.6%)	0.22
	Disability Benefits (Yes)	163 (14.2%)	56 (15.8%)	97 (16.3%)	0.47
Body Mass Index (BMI)					
	Underweight (BMI < 18.5)	6 (0.5%)	3 (0.9%)	6 (1.0%)	0.50
	Normal weight (BMI 18.5–24.9 kg/m^2^)	252 (22.1%)	72 (20.5%)	123 (20.7%)	0.71
	Overweight (BMI 25.0–29.9 kg/m^2^)	389 (34.1%)	129 (36.6%)	220 (37.0%)	0.41
	Obesity (BMI ≥ 30 kg/m^2^)	494 (43.3%)	148 (42.0%)	245 (41.2%)	0.70
Clinical chronic disease (Yes)					
	Hypertension (HAS)	762 (66.3%)	213 (60.2%)	416 (69.8%)	0.01
	Diabetes	356 (31.0%)	96 (27.1%)	172 (28.9%)	0.32
	Stroke	129 (11.2%)	32 (9.0%)	57 (9.6%)	0.36
	Asthma	18 (1.6%)	1 (0.3%)	8 (1.3%)	0.17
	Chronic Obstructive Pulmonary Disease (COPD)	237 (20.6%)	63 (17.8%)	141 (23.7%)	0.09
Medication Profile: Psychotropic and Analgesic Agents					
Psychotropic_medication_use (Yes)					
	Use of anticonvulsants	602 (52.4%)	191 (54.0%)	361 (60.6%)	0.00
	Use of tricyclic/dual antidepressants	568 (49.4%)	201 (56.8%)	280 (47.0%)	0.01
	Use of benzodiazepines	146 (12.7%)	47 (13.3%)	138 (23.2%)	<0.001
	Use of zolpidem	92 (8.0%)	27 (7.6%)	61 (10.2%)	0.22
Analgesic use (Yes)					
	Non-opioid analgesics (≥2 types)	831 (72.3%)	268 (75.7%)	460 (77.2%)	0.07
	Opioid use (any)	552 (48.0%)	191 (54.0%)	320 (53.7%)	0.03
	Tramadol use	413 (35.9%)	149 (42.1%)	250 (41.9%)	0.01
	Morphine use	58 (5.0%)	13 (3.7%)	35 (5.9%)	0.32
	≥1 pain-related medications	260 (22.6%)	82 (23.2%)	169 (28.4%)	0.02
Assessment of care received (Yes)					
Fibromyalgia specialist wait time—consultation schedule (ys)					
	Less than one	628 (54.7%)	194 (54.8%)	331 (55.5%)	0.94
	One to three	250 (21.8%)	86 (24.3%)	123 (20.6%)	0.41
	Four to five	66 (5.7%)	17 (4.8%)	37 (6.2%)	0.66
	More than five reference	204 (17.8%)	57 (16.1%)	105 (17.6%)	0.76
Global treatment quality assessment (0 to 10)		4.99 (3. 91)	5.03 (3.11)	5.06 (3.41)	0.91
Treatment Engagement and Adherence					
	Collaborative treatment decision-making	892 (77.6%)	285 (80.5%)	472 (79.2%)	0.46
	Patient input on prescribed treatment	882 (76.8%)	276 (78.0%)	470 (78.9%)	0.59
	Adherence to recommended treatment plan	188 (16.4%)	49 (13.8%)	99 (16.6%)	0.47
	Self-initiated medication interruption	221 (10.5%)	45 (13.7%)	82 (13.8%)	0.11
	Frequent medication forgetfulness	134 (11.7%)	46 (13.0%)	88 (14.8%)	0.18
	Consistent medication uses as prescribed	965 (84.0%)	300 (84.7%)	519 (87.1%)	0.22
	Exact adherence to prescribed dosage	1028 (89.5%)	309 (87.3%)	514 (86.2%)	0.11

Groups reflect cumulative exposure profiles and are not statistically independent by construction.

**Table 2:** Fibromyalgia Severity, Pain Impact, Psychological Measures, and Adversity Profiles according to cumulative abuse exposure (no abuse, one type of abuse, and two or more types of abuse). Data area presented as mean (SD), median (interquartile) or % (*n* = 2099).

		Cumulative Abuse Exposure			
Variable		No History of Abuse (*n* = 1149)	One Type of Abuse (*n* = 354)	Two or More Types of Abuse (*n* = 596)	*p*-Value
Fibromyalgia Symptom Severity and Diagnostic Criteria (ACR 2016) (Wolfe et al., 2016)					
Widespread pain index (WPI)		10.75 (3.26)	10.55 (3.29)	10.72 (3.37)	0.60
Symptom Severity Score (SSS)		9.66 (1.62)	9.73 (1.60)	10.06 (1.43)	<0.001
Fibromyalgia Severity (FS)—(WPI score plus SSS)		20.40 (3.92)	20.27 (3.91)	20.77 (4.03)	0.09
Pain conditions and impact on daily life					
Diagnosis of other pain conditions					
	Musculoskeletal (Yes)	565 (49.2%)	161 (45.5%)	322 (54.0%)	0.029
	Neuropathic Pain (Yes)	237 (20.6%)	59 (16.7%)	166 (27.9%)	<0.001
Pain intensity—last 7 days (NPS 0–10)		7.94 (1.57)	7.83 (1.58)	8.02 (1.54)	0.19
Pain interfered with enjoyment of life (NPS 0–10)		7.81 (2.30)	7.74 (2.36)	8.16 (2.13)	0.00
Pain interfered with activities (NPS 0–10)		7.93 (2.09)	7.94 (1.94)	8.15 (1.93)	0.08
During the past three months, disability due to pain in daily activities?					
	Some days	396 (34.50%)	124 (35.1%)	177 (29.7%)	0.17
	Most days	752 (65.4%)	229 (64.9%)	418 (70.3%)	0.18
Psychological, Kinesiphobia and Central Sensitization Domains					
Tampa Scale for Kinesiophobia (TSK)		45.47 (8.21)	45.12 (8.20)	47.54 (7.83)	<0.001
Central Sensitization Inventory (CSI)		67.39 (11.91)	68.06 (10.86)	72.50 (10.99)	<0.001
Pain Catastrophizing Scale (PCS)					
	PCS—Magnification	8.10 (3.02)	8.22 (2.79)	8.95 (2.76)	<0.001
	PCS—Helplessness	16.07 (5.10)	16.25 (4.78)	17.62 (4.59)	<0.001
	PCS—Rumination	11.93 (3.19)	12.16 (3.07)	12.68 (2.91)	<0.001
	PCS—Total score	36.11 (10.56)	36.63 (9.91)	39.26 (9.57)	<0.001
History of psychiatric diagnosis (Yes)		509 (44.3%)	156 (44.1%)	364 (61.1%)	<0.001
	Major depression disorders	415 (36.1%)	132 (37.3%)	314 (52.7%)	<0.001
	Anxiety disorder	398 (34.6%)	125 (35.3%)	302 (50.7%)	<0.001
	Bipolar disorder	110 (9.6%)	22 (6.2%)	76 (12.8%)	0.004
	Panic disorder	110 (9.6%)	23 (6.5%)	99 (16.6%)	<0.001
	Post-traumatic stress disorder (PTSD)	53 (4.6%)	15 (4.2%)	73 (12.2%)	<0.001
Number of psychiatric comorbidities (excluding depression)		0.94 (1.22)	0.89 (1.17)	1.45 (1.41)	<0.001
Patient Health Questionnaire-9 (PHQ-9)		16.75 (6.14)	17.03 (5.92)	18.84 (5.78)	<0.001
Life adverse events					
	Persistent physical aggression (Yes)	160 (13.9%)	57 (16.1%)	282 (47.3%)	<0.001
	Persistent verbal aggression (Yes)	131 (11.4%)	4 (1.1%)	596 (100.0%)	<0.001
	Persistent emotional aggression (Yes)	360 (31.3%)	122 (34.5%)	596 (100.0%)	<0.001
	Sexual abuse (Yes)	97 (8.4%)	46 (13.0%)	222 (37.2%)	<0.001
Fibromyalgia_onset_related_to_major_life_event (Yes)		269 (23.4%)	228 (64.4%)	596 (100.0%)	<0.001
Duration of fibromyalgia (ys)		15.15 (11.83)	14.18 (11.49)	15.86 (11.16)	0.09
Age at onset of fibromyalgia (ys)		34.56 (12.11)	32.93 (11.37)	31.76 (11.78)	<0.001
Age at onset of fibromyalgia categories (ys)					
	Onset before 18 ys	88 (7.9%)	29 (8.5%)	75 (13.4%)	0.001
	19–25 ys	164 (14.8%)	71 (20.8%)	101 (18.0%)	0.021
	26–40 ys	501 (45.2%)	147 (43.1%)	245 (43.7%)	0.723
	>40 ys	355 (32.0%)	94 (27.6%)	140 (25.0%)	0.008
Physical activities performed regularly		572 (49.8%)	178 (50.3%)	309 (51.8%)	0.42

Groups reflect cumulative exposure profiles and are not statistically independent by construction. Non-depressive psychiatric comorbidity (cumulative count): cumulative number of self-reported lifetime diagnoses of anxiety disorders, bipolar disorder, panic disorder, and post-traumatic stress disorder (PTSD).

**Table 3:** Dose–response associations between cumulative abuse exposure and fibromyalgia-related symptom severity and functional impact. (*n* = 2099).

Fatigue Symptoms	Β	SE	*p*-Value	
Cumulative abuse (range 0–4)	0.02	(0.01, 0.04)	0.004	Adjusted R^2^ = 0.06
Age (ys)	0.00	(−0.00, 0.00)	0.131	
Education level (ys)	0.20	0.12 to 0.29	<0.001	
Disability due to pain in daily activities	0.00	−0.00 to 0.00	0.689	
Body mass index (BMI)	0.20	0.12 to 0.29	<0.001	
Cognitive dysfunction symptoms				Adjusted R^2^ = 0.04
Cumulative abuse (range 0–4)	0.05	(0.03, 0.07)	0.000	
Age (ys)	−0.00	(−0.01, −0.00)	0.006	
Education level (ys)	0.20	0.09 to 0.32	<0.001	
Disability due to pain in daily activities	0.00	(−0.02, 0.02)	0.998	
Body mass index (BMI)	0.01	(−0.02, 0.05)	0.521	
Waking unrefreshed				Adjusted R^2^ = 0.03
Cumulative abuse (range 0–4)	0.03	(0.01, 0.05)	0.001	
Age (ys)	−0.00	(−0.00, 0.00)	0.755	
Education level (ys)	0.17	0.08 to 0.26	<0.001	
Disability due to pain in daily activities	0.01	(−0.01, 0.02)	0.530	
Body mass index (BMI)	0.03	(0.00, 0.06)	0.038	
Fibromyalgia Impact Questionnaire (FIQ)				Adjusted R^2^ = 0.14
Cumulative abuse (range 0–4)	1.33	(0.92, 1.74)	0.000	
Age (ys)	−0.07	(−0.13, −0.02)	0.011	
Education level (ys)	7.99	5.98 to 10.01	<0.001	
Disability due to pain in daily activities	0.99	(0.56, 1.41)	0.000	
Body mass index (BMI)	1.67	(0.98, 2.36)	0.000	

β represents the change in outcome per one-unit increase in cumulative abuse (range 0–4). β represents the standardized regression coefficient; SE = standard error. Model R^2^ indicates the proportion of variance in each symptom domain explained by adversity indicators after covariate adjustment.

**Table 4:** Mediation analysis: Regression model for four clinical domains: symptoms of cognitive dysfunction, fatigue, non-restorative sleep, and global fibromyalgia impact (FIQ) (*n* = 2076).

	a_coef	b_coef	c_total	c_prime	indirect_ab	Proportion Mediated
Symptoms of cognitive dysfunction						
Major life loss (Yes)	−0.325	−0.207	0.101	0.033	0.067	0.667
Persistent physical aggression (Yes)	−0.976	−0.229	0.113	−0.110	0.223	1.969
Persistent verbal aggression (Yes)	−0.493	−0.205	0.137	0.036	0.101	0.739
Persistent emotional aggression (Yes)	−0.425	−0.207	0.107	0.019	0.088	0.820
Sexual abuse (Yes)	−0.565	−0.211	0.097	−0.022	0.119	1.228
Two types of abuse	−0.273	−0.212	0.053	−0.005	0.058	1.090
Three or more types of abuse	−0.250	−0.204	0.081	0.030	0.051	0.630
Fatigue						
Major life loss (Yes)	−0.325	−0.255	0.034	−0.049	0.083	2.418
Persistent physical aggression (Yes)	−0.976	−0.284	0.087	−0.190	0.277	3.182
Persistent verbal aggression (Yes)	−0.493	−0.260	0.046	−0.082	0.128	2.806
Persistent emotional aggression (Yes)	−0.425	−0.258	0.036	−0.074	0.110	3.067
Sexual abuse (Yes)	−0.565	−0.259	0.044	−0.103	0.146	3.343
Two types of abuse	−0.273	−0.275	0.024	−0.051	0.075	3.163
Three or more types of abuse	−0.250	−0.258	0.024	−0.041	0.064	2.692
Non-restorative sleep						
Major life loss (Yes)	−0.325	−0.169	0.048	−0.006	0.055	1.133
Persistent physical aggression (Yes)	−0.976	−0.179	0.116	−0.059	0.174	1.506
Persistent verbal aggression (Yes)	−0.493	−0.170	0.065	−0.019	0.084	1.291
Persistent emotional aggression (Yes)	−0.425	−0.174	0.018	−0.056	0.074	4.049
Sexual abuse (Yes)	−0.565	−0.169	0.081	−0.015	0.096	1.187
Two types of abuse	−0.273	−0.177	0.030	−0.019	0.048	1.624
Three or more types of abuse	−0.250	−0.169	0.037	−0.005	0.042	1.130
Fibromyalgia Impact Questionnaire (FIQ)						
Major life loss (Yes)	−0.325	−6.802	1.709	−0.503	2.212	1.294
Persistent physical aggression (Yes)	−0.976	−7.542	2.948	−4.416	7.364	2.498
Persistent verbal aggression (Yes)	−0.493	−6.810	2.919	−0.440	3.359	1.151
Persistent emotional aggression (Yes)	−0.425	−6.803	2.500	−0.392	2.893	1.157
Sexual abuse (Yes)	−0.565	−6.823	3.090	−0.768	3.859	1.249
Two types of abuse	−0.273	−7.060	1.297	−0.629	1.926	1.485
Three or more types of abuse	−0.250	−6.770	1.642	−0.050	1.691	1.030

a_coef—standardized effect of abuse on the mediator (SOC). b_coef—standardized effect of the mediator (SOC) on the outcome, controlling for abuse. c_total—total effect of abuse on the outcome. c_prime—direct effect of abuse on the outcome, controlling for SOC. indirect_ab—indirect (mediated) effect of abuse through SOC (a × b). Proportion mediated—proportion of the total effect mediated by SOC.

**Table 5:** Moderation analysis: regression models for symptoms of cognitive dysfunction, fatigue, non-restorative sleep, and global fibromyalgia impact (FIQ) (*n* = 2076).

Adversity Indicator		Beta-Interaction	*p*-Value
Outcome: symptoms of cognitive dysfunction			
	Major life loss (Yes)	0.023	0.401
	Persistent physical aggression (Yes)	0.003	0.940
	Persistent verbal aggression (Yes)	0.040	0.167
	Persistent emotional aggression (Yes)	0.035	0.215
	Sexual abuse (Yes)	0.035	0.334
	Two types of abuse	0.017	0.099
	Three or more types of abuse	0.017	0.282
Outcome: Fatigue			
	Major life loss (Yes)	0.052	0.009
	Persistent physical aggression (Yes)	0.039	0.139
	Persistent verbal aggression (Yes)	0.060	0.004
	Persistent emotional aggression (Yes)	0.057	0.005
	Sexual abuse (Yes)	0.057	0.027
	Two types of abuse	0.024	0.001
	Three or more types of abuse	0.024	0.028
Outcome: Non-restorative sleep			
	Major life loss (Yes)	0.027	0.228
	Persistent physical aggression (Yes)	0.050	0.087
	Persistent verbal aggression (Yes)	0.031	0.179
	Persistent emotional aggression (Yes)	0.015	0.496
	Sexual abuse (Yes)	0.044	0.121
	Two types of abuse	0.012	0.133
	Three or more types of abuse	0.014	0.265
Outcome: Fibromyalgia Impact Questionnaire (FIQ)			
	Major life loss (Yes)	0.804	0.100
	Persistent physical aggression (Yes)	1.154	0.072
	Persistent verbal aggression (Yes)	1.583	0.002
	Persistent emotional aggression (Yes)	1.437	0.004
	Sexual abuse (Yes)	2.106	0.001
	Two types of abuse	0.751	0.000
	Three or more types of abuse	0.569	0.039

β-interaction represents the coefficient of the adversity × SOC interaction term in the regression model. Positive β-interaction values indicate that adversity–symptom associations are stronger among individuals with lower SOC and attenuated among those with higher SOC, consistent with a buffering (protective) moderating effect of SOC. Non-significant interaction coefficients indicate absence of moderation.

## Data Availability

Data Availability Statement: The datasets generated and/or analyzed during the current study are available from the corresponding author upon reasonable request. All data are fully de-identified to protect participant confidentiality. Requests for data access can be directed to: wcaumo@hcpa.edu.br.

## Supplementary Materials

The following supporting information can be downloaded at: https://www.mdpi.com/article/10.3390/ejihpe16030045/s1. Figure S1. Construction and Validation of the SOC Composite. Table S1. Variables included in the construction of SOC domains. Table S2. Correlations between SOC domains and core clinical outcomes (FIQ, fatigue, non-restorative sleep, and cognitive symptoms) (*n* = 2099). Table S3. Associations between Adversity Indicators and Symptom Outcomes (*n* = 2099). Table S4. Interaction (Moderation) and Mediation Effects Linking Adversity to Clinical Outcomes (*n* = 2099).

## Abbreviations

ACR	American College of Rheumatology
AUDIT-C	Alcohol Use Disorders Identification Test—Consumption
BMI	Body Mass Index
COPD	Chronic Obstructive Pulmonary Disease
CSI	Central Sensitization Inventory
FIQ	Fibromyalgia Impact Questionnaire
FM	Fibromyalgia
FS	Fibromyalgia Severity (WPI + SSS)
HCPA	Hospital de Clínicas de Porto Alegre
ICF	Informed Consent Form
IRB	Institutional Review Board
NPS	Numeric Pain Scale
NSAIDs	Non-Steroidal Anti-Inflammatory Drugs
PCS	Pain Catastrophizing Scale
PHQ-9	Patient Health Questionnaire-9
PTSD	Post-Traumatic Stress Disorder
REDCap	Research Electronic Data Capture
SOC	Sense of Coherence
SSRI	Selective Serotonin Reuptake Inhibitor
SSS	Symptom Severity Scale
STROBE	Strengthening the Reporting of Observational Studies in Epidemiology
TSK	Tampa Scale for Kinesiophobia
UFRGS	Universidade Federal do Rio Grande do Sul
WPI	Widespread Pain Index
