# Supplementary material for: Sense of Coherence in the Trauma–Fibromyalgia Relationship: Mediation and Moderation Findings from a 2099-Participant Cohort
Source: Eur J Investig Health Psychol Educ. 2026 Mar 23;16(3):45. doi: 10.3390/ejihpe16030045 (PMC13025946; doi:10.3390/ejihpe16030045)
Supplement: Supplementary file 1 [file ejihpe-16-00045-s001.zip › ejihpe-4134259-supplementary.pdf]

Supplementary Material

Supplementary Methods — Construction and Validation of the SOC Composite.

To operationalize sense of coherence (SOC) within a biopsychosocial framework, domain scores were constructed using standardized proxy indicators reflecting emotional burden, functional coping capacity, healthcare engagement, and illness interpretation. All variables were standardized (z-scores) prior to aggregation. Domain scores were calculated as the mean of standardized indicators within each domain, and the global SOC composite was calculated as the mean of the three domain scores. This supplementary material details the variable composition, transformation steps, and correlation structure supporting discriminant validity from outcome measures.

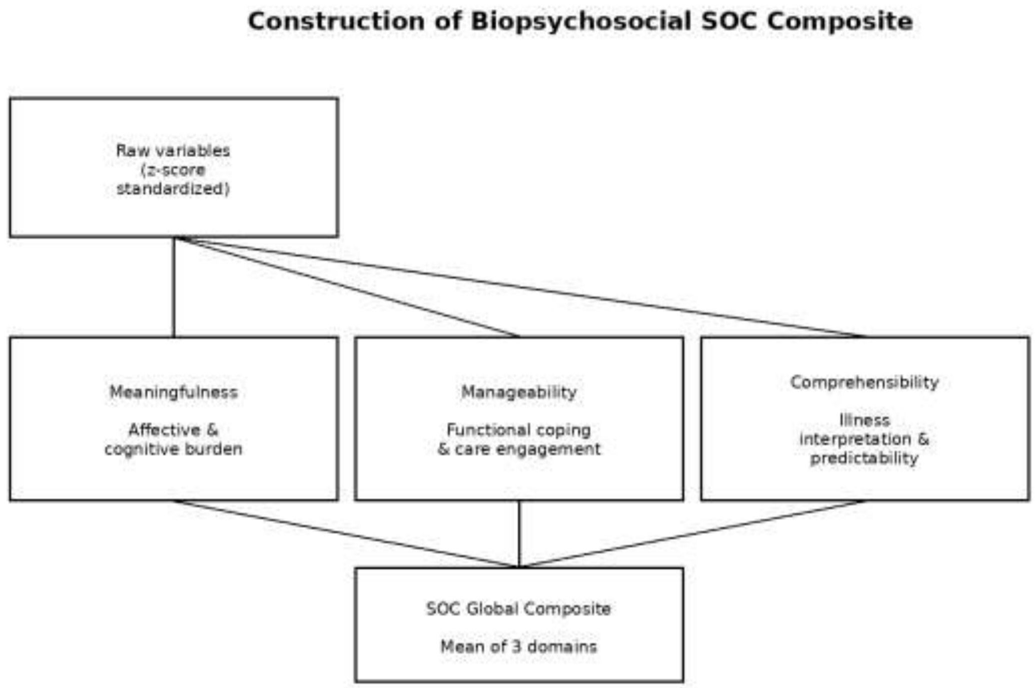

Figure S1: Raw questionnaire and clinical variables were standardized (z-scores) and aggregated into three conceptual domains reflecting the biopsychosocial components of sense of coherence (SOC): Meaningfulness, Manageability, and Comprehensibility.

a) The meaningfulness domain was constructed from affective and cognitive-emotional burden indicators, including pain catastrophizing (total PCS score), depressive symptom severity assessed exclusively by the PHQ-9 total score, and psychiatric diagnosis burden reflecting non-depressive psychiatric comorbidity, operationalized as a cumulative count

of self-reported lifetime diagnoses of anxiety disorders, bipolar disorder, panic disorder, and post-traumatic stress disorder (PTSD). These variables represent affective appraisal and emotional valuation of illness experiences.

b) The Manageability domain incorporated indicators of functional coping resources, behavioral engagement, and healthcare-related support, including work status (categorical variable: unemployed, employed, student, caregiver, self-employed, retired, and receiving disability benefits), physical activity (regular participation in physical activities). Medication-adherence behaviors (consistent medication use as prescribed, adherence to the recommended treatment plan, frequent medication forgetfulness [reverse-coded], and self-initiated medication interruption [reverse-coded]), treatment engagement and perceived control (collaborative treatment decision-making, patient input on prescribed treatment, waiting time for fibromyalgia specialist consultation, and global treatment quality assessment), and complexity of pharmacological treatment (number of medication classes in use). These indicators collectively represent perceived availability of internal and external resources and capacity to cope with health-related demands

c) The **Comprehensibility** domain reflected illness interpretation and symptom predictability, incorporating indicators of bodily threat appraisal and symptom coherence, including diagnosis of other pain conditions (musculoskeletal and neuropathic pain), central sensitization (Central Sensitization Inventory, CSI score), kinesiophobia (Tampa Scale of Kinesiophobia, TSK score), perceived linkage between symptoms and physical aggression, and age at fibromyalgia onset (before 18 years, 19–25 years, 26–40 years, and >40 years).

Domain scores were computed as the mean of standardized indicators, and the global SOC composite was calculated as the mean of the three domain scores. Equal weighting was applied across indicators and domains to avoid dominance of any single measurement source. Objective adversity indicators (e.g., cumulative abuse and specific traumatic events) were not included in the SOC composite.

Table S1 summarizes the theory-driven construction of the Sense of Coherence (SOC) surrogate, aligned with Antonovsky's salutogenic framework, including the three theoretical domains and the clinical, psychological, and health care indicators used to operationalize each domain.

Table S1. Variables included in the construction of SOC domains.

SOC Domain	Conceptual Dimension	Indicators Included
Meaningfulness	Affective and cognitive-emotional valuation of symptoms and life context	• Pain catastrophizing (PCS total score)<br>• Depressive symptoms (PHQ-9 score)<br>• Psychiatric diagnosis burden (Psychiatric diagnosis burden reflected non-depressive psychiatric comorbidity and was modeled separately from depressive symptoms, which were assessed using the PHQ-9 total score).
Comprehensibility	Cognitive understanding, symptom predictability, and bodily threat appraisal	• Diagnosis of other pain conditions (musculoskeletal pain)<br>• Diagnosis of neuropathic pain<br>• Central sensitization (CSI score)

		• Kinesiophobia (TSK score)<br>• Perceived linkage between symptoms and physical aggression<br>• Age at fibromyalgia onset (before 18, 19–25, 26–40, >40 years)
Manageability	Perceived availability of resources and capacity to cope with health demands	• Work status (unemployed, employed, student, caregiver, self-employed, retired, receiving disability benefits)<br>• Physical activity (regular engagement in physical activities)<br>• Medication-adherence behaviors: medication use as prescribed adherence to treatment plan medication forgetfulness (reverse-coded) self-initiated medication interruption (reverse-coded)<br>• Treatment engagement and perceived control: collaborative treatment decision-making patient input on prescribed treatment waiting time for specialist consultation global treatment quality assessment<br>• Complexity of pharmacological treatment (number of medication classes)

Note: A dedicated SOC questionnaire was not administered. Instead, a theory-driven SOC surrogate was constructed using available indicators reflecting each theoretical domain of Antonovsky's model.

Correlations between clinical outcomes and SOC

This table S2 presents **correlations between the main clinical** outcomes (FIQ total score, fatigue, non-restorative sleep, and cognitive symptoms) and the global SOC composite, as well as its three domains (Meaningfulness, Manageability, and Comprehensibility). SOC scores were constructed from standardized variables (z-scores), with domain scores calculated as the mean of standardized indicators within each domain and the SOC total score calculated as the mean of the three domains. Observed correlations were of low to moderate magnitude, particularly for the Meaningfulness domain, indicating conceptually plausible associations between emotional/cognitive burden and symptom severity. Importantly, no high correlations ($r \geq 0.70$) were observed, suggesting absence of severe multicollinearity or mathematical redundancy between SOC constructs and clinical outcomes, thereby supporting discriminant validity of the SOC composite in relation to symptom severity and functional impact measures.

Table S2. Correlations between SOC domains and core clinical outcomes (FIQ, fatigue, non-restorative sleep, and cognitive symptoms) (n=2099).

Outcome	SOC_total	SOC_Meaningfulness	SOC_Manageability	SOC_Comprehensibility
Fibromyalgia Impact Questionnaire (FIQ)	0.37*	0.36*	0.33*	0.20**
Fatigue	0.42*	0.44*	0.21*	0.01
Non-restorative sleep	0.27*	0.35*	0.15*	0.08
Cognitive symptoms	0.26*	0.34*	0.11*	0.12

**P<0.001; **P<0.01, Sense of Coherence (SOC)*

Supplementary Analyses: Individual Adversity Indicators

Supplementary Table S3 presents adjusted regression models examining the associations between individual adversity indicators (major loss events, persistent physical aggression, verbal aggression, emotional aggression, and sexual abuse) and clinical outcomes. These analyses were conducted to explore whether specific adverse experiences showed differential associations with symptom domains beyond the cumulative abuse effect. Overall, individual adversity indicators showed small but consistent associations, particularly for cognitive dysfunction and non-restorative sleep, supporting the cumulative adversity approach used in the primary analyses

Table S3. Associations between Adversity Indicators and Symptom Outcomes (n=2099).

**Fatigue symptoms**				
**Adversity indicator**	**β**	**SE**	**p-value**	
Major loss events	0.01	(-0.04, 0.06)	0.681	Adjusted R^2 = 0.06
Persistent physical aggression	0.07	(0.01, 0.13)	0.018	
Verbal aggression	0.02	(-0.04, 0.08)	0.532	
Emotional aggression	-0.01	(-0.07, 0.06)	0.864	
Sexual abuse	0.02	(-0.05, 0.08)	0.571	
Age (ys)	0.00	(-0.00, 0.00)	0.136	
Education level (ys)	0.20	0.12 to 0.29	<0.001	
Disability due to pain in daily activities	0.02	(-0.00, 0.03)	0.080	
Body mass index (BMI)	0.05	(0.02, 0.08)	0.000	
**Cognitive dysfunction symptoms**				Adjusted R^2=0.04
Major loss events	0.04	(-0.03, 0.11)	0.266	

	Persistent physical aggression	0.05	(0.02, 0.13)	0.01	
	Verbal aggression	0.10	(0.01, 0.18)	0.022	
	Emotional aggression	0.01	(0.08, 0.09)	0.031	
	Sexual abuse	0.02	(-0.06, 0.10)	0.668	
Age (ys)		-0.00	(-0.01, -0.00)	0.006	
Education level (ys)		0.20	0.09 to 0.32	<0.001	
Disability due to pain in daily activities		-0.00	(-0.02, 0.02)	0.978	
Body mass index (BMI)		0.01	(-0.02, 0.05)	0.518	
Waking unrefreshed					Adjusted R^2=0.03
	Major loss events	0.03	(-0.02, 0.09)	0.234	
	Persistent physical aggression	0.10	(0.04, 0.15)	0.001	
	Verbal aggression	0.07	(-0.00, 0.13)	0.051	
	Emotional aggression	-0.08	(-0.14, -0.01)	0.019	
	Sexual abuse	0.04	(-0.02, 0.11)	0.213	
Age (ys)		0.04	(-0.02, 0.11)	0.213	
Education level (ys)		0.17	0.08 to 0.26	<0.001	
Disability due to pain in daily activities		-0.00	(-0.00, 0.00)	0.765	
Body mass index (BMI)		0.00	(-0.01, 0.02)	0.626	
Fibromyalgia Impact Questionnaire (FIQ)					**Adjusted R^2=0.15**
	Major loss events	-0.05	(-0.06, 0.02)	0.939	
	Persistent physical aggression	1.45	(1.27, 1.37)	0.045	
	Verbal aggression	1.88	(0.03, 2.87)	0.021	
	Emotional aggression	0.63	(0.29, 3.47)	0.432	
	Sexual abuse	1.53	(0.95, 2.21)	0.041	
Age (ys)		-0.07	(-0.05, -3.12)	0.011	
Education level (ys)		8.02	6.00 to 10.04	<0.001	
Disability due to pain in daily activities		0.98	(0.13, 1.2)	0.000	
Body mass index (BMI)		1.68	(0.56, 1.41)	0.000	

All models were adjusted for age, education, BMI, disability due to pain in daily activities.

β represents the standardized regression coefficient; SE = standard error; ns = non-significant. Model R^2 indicates the proportion of variance in each symptom domain explained by adversity indicators after covariate adjustment.

Table S4- Interaction (Moderation) and Mediation Effects Linking Adversity to Clinical Outcomes (n=2099).

A. Moderation Effects		
**Outcomes variables – Clinical variable and interaction variables**	**β_interaction**	**p-value**
**Symptoms of cognitive dysfunction**		
Scheduled consultation × Persistent physical aggression	-0.46	.022
Scheduled consultation × Persistent verbal aggression	-0.38	.036
Dual-action antidepressants × Persistent physical aggression	0.21	.002
Antidepressant tricyclic antidepressants × Persistent physical aggression	0.12	.023
Dual-action antidepressants × Other abuse types	0.05	.034
**Fatigue**		
Scheduled consultation ×Emotional aggression	0.36	.004
Stroke ×Sexual abuse	0.19	.034
**Fibromyalgia Impact Questionnaire (FIQ)**		
Stroke ×Major life loss	4.94	.007
Stroke ×. Sexual abuse	5.15	.023
Zolpidem ×Persistent physical aggression	-4.55	.041

B. Mediation Effects						
**Fatigue**						
Mediator	Adversity	a-path	p(a)	**b-path**	**p(b)**	**Indirect(ab)**
Hypertension (HAS)	Physical aggression	0.07	.006	0.12	<.001	0.01
Hypertension (HAS)	Emotional aggression	0.05	.023	0.12	<.001	0.006
Hypertension (HAS)	Cumulative abuse	0.02	.009	0.12	<.001	0.002
Diabetes	Major life loss	-0.05	.01	0.08	<.001	0.004
Non-restorative sleep						
Hypertension (HAS)	Cumulative abuse	-0.03	0.01	0.07	<.01	≤0.01
Outcome: Fibromyalgia Impact Questionnaire (FIQ)						
Hypertension (HAS)	Physical aggression	0.07	.006	4.03	<.001	0.27
Hypertension (HAS)	Emotional aggression	0.05	.023	3.98	<.001	0.19
Hypertension (HAS)	Cumulative abuse	0.02	.009	4.03	<.001	0.08
Diabetes	Major life loss	-0.05	.01	1.47	.017	0.07

Table S4A. *β_interaction denotes the coefficient of the interaction term (Adversity × Moderator). Significant values indicate that the slope of the adversity–the moderator modifies outcome relationship."*

Table S4B. *a-path: effect of adversity on the mediator (adjusted for the predictor e.g., hypertension or diabetes);*

b-path: effect of the mediator on the clinical outcome (fatigue, non-restorative sleep, or FIQ), adjusted for adversity; p(a) and p(b): corresponding significance values."
